# Poly[[tetra­aqua­tetra­kis­[μ_3_-5-(pyridine-4-carboxamido)­isophthalato]nickel(II)diterbium(III)] tetra­hydrate]

**DOI:** 10.1107/S1600536813014876

**Published:** 2013-06-08

**Authors:** Yi-Fang Deng, Man-Sheng Chen, Chun-Hua Zhang, Xue Nie

**Affiliations:** a Key Laboratory of Functional Organometallic Materials, Hengyang Normal University, Department of Chemistry and Materials Science, Hengyang, Hunan 421008, People’s Republic of China

## Abstract

In the title compound, {[NiTb_2_(C_14_H_8_N_2_O_5_)_4_(H_2_O)_4_]·4H_2_O}_*n*_, the Tb^III^ ion is coordinated by one water mol­ecule and seven O atoms from four 5-(pyridine-4-carboxamido)­isophthalate (*L*) ligands in a distorted square-anti­prismatic arrangement, while the Ni^II^ ion, lying on an inversion center, is six-coordinated in an octa­hedral geometry by two pyridine N atoms, two carboxyl­ate O atoms and two water mol­ecules. One *L* ligand bridges two Tb^III^ ions and one Ni^II^ ion through two carboxyl­ate groups and one pyridine N atom. The other *L* ligand bridges two Tb^III^ ions and one Ni^II^ ion through two carboxyl­ate groups, while the uncoordinating pyridine N atom is hydrogen bonded to an adjacent coordinating water mol­ecule. Extensive O—H⋯O, N—H⋯O and O—H⋯N hydrogen bonds play an important role in stabilizing the crystal structure.

## Related literature

For background to hetero-metallic complexes, see: Gu & Xue (2006 ▶); Liang *et al.* (2000 ▶); Prasad *et al.* (2007 ▶); Zhao *et al.* (2003 ▶, 2004 ▶). For related structures, see: Chen *et al.* (2011 ▶); Deng *et al.* (2011 ▶).
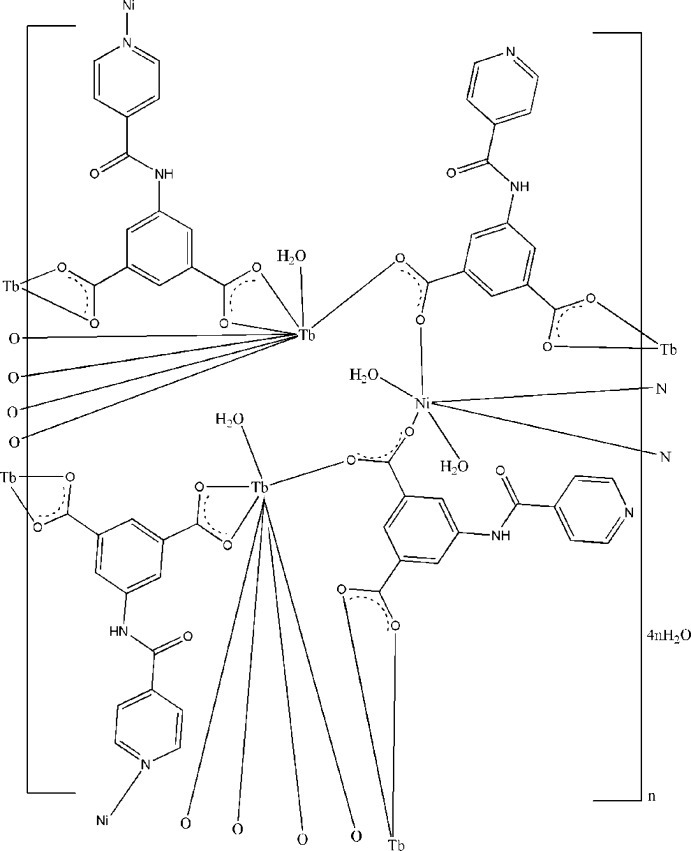


## Experimental

### 

#### Crystal data

[NiTb_2_(C_14_H_8_N_2_O_5_)_4_(H_2_O)_4_]·4H_2_O
*M* *_r_* = 1657.57
Triclinic, 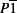

*a* = 10.2347 (12) Å
*b* = 10.8608 (13) Å
*c* = 13.7452 (17) Å
α = 79.053 (2)°
β = 78.745 (1)°
γ = 86.326 (2)°
*V* = 1470.7 (3) Å^3^
*Z* = 1
Mo *K*α radiation
μ = 2.80 mm^−1^
*T* = 293 K
0.22 × 0.16 × 0.08 mm

#### Data collection

Bruker APEX CCD diffractometer
Absorption correction: multi-scan (*SADABS*; Bruker, 2001 ▶) *T*_min_ = 0.578, *T*_max_ = 0.807
7345 measured reflections
5088 independent reflections
4486 reflections with *I* > 2σ(*I*)
*R*_int_ = 0.045

#### Refinement

*R*[*F* ^2^ > 2σ(*F* ^2^)] = 0.033
*wR*(*F* ^2^) = 0.074
*S* = 1.03
5088 reflections
434 parameters
1 restraint
H atoms treated by a mixture of independent and constrained refinement
Δρ_max_ = 1.24 e Å^−3^
Δρ_min_ = −0.85 e Å^−3^

### 

Data collection: *SMART* (Bruker, 2007 ▶); cell refinement: *SAINT* (Bruker, 2007 ▶); data reduction: *SAINT*; program(s) used to solve structure: *SHELXTL* (Sheldrick, 2008 ▶); program(s) used to refine structure: *SHELXTL*; molecular graphics: *XP* in *SHELXTL* and *DIAMOND* (Brandenburg, 1999 ▶); software used to prepare material for publication: *SHELXTL*.

## Supplementary Material

Crystal structure: contains datablock(s) global, I. DOI: 10.1107/S1600536813014876/hy2627sup1.cif

Structure factors: contains datablock(s) I. DOI: 10.1107/S1600536813014876/hy2627Isup2.hkl

Additional supplementary materials:  crystallographic information; 3D view; checkCIF report

## Figures and Tables

**Table 1:** Hydrogen-bond geometry (Å, °)

*D*—H⋯*A*	*D*—H	H⋯*A*	*D*⋯*A*	*D*—H⋯*A*
N1—H1⋯O4*W* ^i^	0.86	2.16	3.000 (6)	166
N3—H3⋯O4^ii^	0.86	2.17	2.958 (6)	153
O1*W*—H1*WA*⋯O6^iii^	0.82	2.24	2.988 (4)	151
O1*W*—H1*WB*⋯O3*W* ^iv^	0.85	2.04	2.763 (6)	143
O2*W*—H2*WA*⋯O3*W* ^v^	0.85 (6)	2.47 (6)	3.117 (7)	134 (5)
O2*W*—H2*WB*⋯N2^vi^	0.85	1.92	2.672 (6)	147
O3*W*—H3*WC*⋯O3^iv^	0.85	1.92	2.736 (5)	159
O3*W*—H3*WD*⋯O8^vii^	0.85	1.98	2.793 (5)	160
O4*W*—H4*WA*⋯O9^viii^	0.85	2.25	3.088 (6)	170
O4*W*—H4*WB*⋯O9^ii^	0.85	2.19	3.034 (5)	172

Symmetry codes: (i) 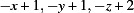; (ii) 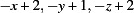; (iii) 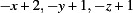; (iv) 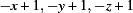; (v) 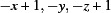; (vi) 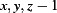; (vii) 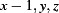; (viii) 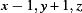.

**Fig. 1.**
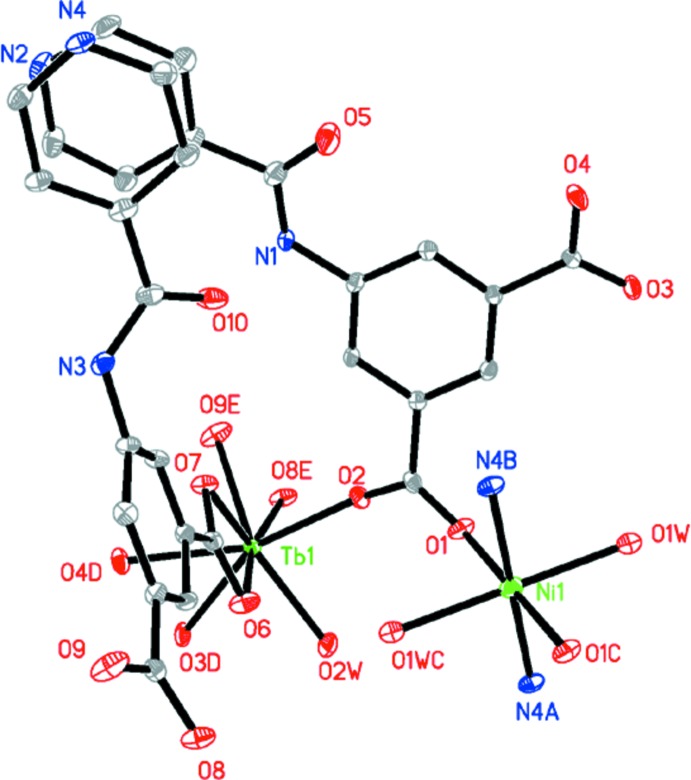
The asymmetric unit of the title compound. Displacement ellipsoids are drawn at the 30% probability level. [Symmetry codes: (A) x, y, -1+z; (B) 2-x, 1-y, 2-z; (C) 2-x, 1-y, 1-z; (D) x, -1+y, z; (E) -1+x, y, z.]

**Fig. 2.**
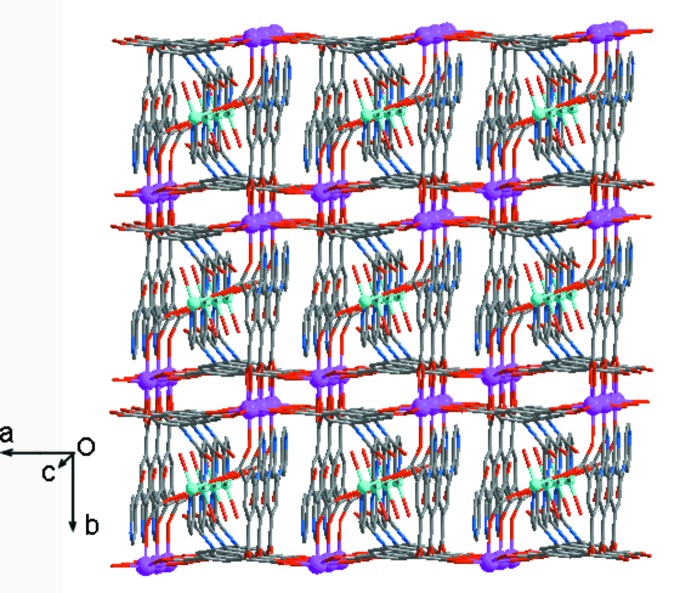
A view showing the three-dimensional network of the title compound.

## supplementary crystallographic information

### Comment

The synthesis and investigation of d-f heterometallic complexes are challenge for chemists and have attracted increasing attention in last few years since the competitive reaction containing d-f metal ions in conjunction with ligands often result in the formation of a mixture of homometallic assemblies rather than heterometallic analogous (Gu & Xue, 2006; Liang *et al.*, 2000; Prasad *et al.*, 2007; Zhao *et al.*, 2003, 2004;). We have recently prepared the title compound, a new transition metal(II)–lanthanide(III) coordination polymer, under hydrothermal conditions.

In the title compound, the Tb^III^ ion is eight-coordinated by seven O atoms from four 5-(pyridine-4-carboxamido)isophthalate (*L*) ligands and one water molecule, forming a distorted square-antiprismatic geometry (Fig. 1). It is interesting that the carboxylate groups of two unique *L* ligands exhibit different coordination modes: one coordinates to two Tb^III^ ions and one Ni^II^ ion using its two carboxylate groups with µ_1_-η^1^:η^1^-chelate and µ_2_-η^1^:η^1^-bis-monodentate coordination modes while the pyridyl group is free of coordination, the other one coordinates to two Tb^III^ ions through the carboxylate groups with µ_1_-η^1^:η^1^-chelate coordination mode and to one Ni^II^ ion through the pyridyl group. Based on the coordination modes of the carboxylate and pyridyl groups, a complicated three-dimensional network is formed (Fig. 2), which is similar to the complexes {[LnCo_0.5_(INAIP)_2_(H_2_O)_2_].2H_2_O}_n_ (Chen *et al.*, 2011; Deng *et al.*, 2011).

### Experimental

A mixture of Tb(NO_3_)_3_.6H_2_O (22.1 mg. 0.05 mmol), H_2_*L* (28.7 mg, 0.1 mmol), NiSO_4_.6H_2_O (13.1 mg, 0.05 mmol), NaOH (6.0 mg, 0.15 mmol), EtOH (4 ml) and H_2_O (6 ml) was heated in a 16 ml capacity Teflon-lined reaction vessel at 453 K for 3 days. The reaction mixture was cooled to room temperature over a period of 48 h. The product was collected by filtration, washed with H_2_O and air-dried.

### Refinement

H atoms bonded to C and N atoms were placed geometrically and refiined as riding atoms, with C—H = 0.93 and N—H = 0.86 Å and with *U*_iso_(H) = 1.2*U*_eq_(C, N). The water H atoms were found from difference Fourier maps and refined with a restraint of O—H = 0.85 (1) Å. In final refinements, these H atoms were refined as riding atoms with *U*_iso_(H) = 1.2*U*_eq_(O). H2*WA* was refined isotropically.

### Crystal data

**Table:**

[NiTb_2_(C_14_H_8_N_2_O_5_)_4_(H_2_O)_4_]·4H_2_O	*Z* = 1
*M**_r_* = 1657.57	*F*(000) = 822
Triclinic, *P*1	*D*_x_ = 1.872 Mg m^−^^3^
Hall symbol: -P 1	Mo *K*α radiation, λ = 0.71073 Å
*a* = 10.2347 (12) Å	Cell parameters from 3746 reflections
*b* = 10.8608 (13) Å	θ = 2.1–24.8°
*c* = 13.7452 (17) Å	µ = 2.80 mm^−^^1^
α = 79.053 (2)°	*T* = 293 K
β = 78.745 (1)°	Block, green
γ = 86.326 (2)°	0.22 × 0.16 × 0.08 mm
*V* = 1470.7 (3) Å^3^	

### Data collection

**Table:**

Bruker APEX CCD diffractometer	5088 independent reflections
Radiation source: fine-focus sealed tube	4486 reflections with *I* > 2σ(*I*)
Graphite monochromator	*R*_int_ = 0.045
φ and ω scans	θ_max_ = 25.0°, θ_min_ = 2.0°
Absorption correction: multi-scan (*SADABS*; Bruker, 2001)	*h* = −12→10
*T*_min_ = 0.578, *T*_max_ = 0.807	*k* = −12→12
7345 measured reflections	*l* = −15→16

### Refinement

**Table:**

Refinement on *F*^2^	Primary atom site location: structure-invariant direct methods
Least-squares matrix: full	Secondary atom site location: difference Fourier map
*R*[*F*^2^ > 2σ(*F*^2^)] = 0.033	Hydrogen site location: inferred from neighbouring sites
*wR*(*F*^2^) = 0.074	H atoms treated by a mixture of independent and constrained refinement
*S* = 1.03	*w* = 1/[σ^2^(*F*_o_^2^) + (0.0233*P*)^2^] where *P* = (*F*_o_^2^ + 2*F*_c_^2^)/3
5088 reflections	(Δ/σ)_max_ = 0.013
434 parameters	Δρ_max_ = 1.24 e Å^−^^3^
1 restraint	Δρ_min_ = −0.85 e Å^−^^3^

### Special details

**Table:**

Geometry. All e.s.d.'s (except the e.s.d. in the dihedral angle between two l.s. planes) are estimated using the full covariance matrix. The cell e.s.d.'s are taken into account individually in the estimation of e.s.d.'s in distances, angles and torsion angles; correlations between e.s.d.'s in cell parameters are only used when they are defined by crystal symmetry. An approximate (isotropic) treatment of cell e.s.d.'s is used for estimating e.s.d.'s involving l.s. planes.
Refinement. Refinement of *F*^2^ against ALL reflections. The weighted *R*-factor *wR* and goodness of fit *S* are based on *F*^2^, conventional *R*-factors *R* are based on *F*, with *F* set to zero for negative *F*^2^. The threshold expression of *F*^2^ > σ(*F*^2^) is used only for calculating *R*-factors(gt) *etc*. and is not relevant to the choice of reflections for refinement. *R*-factors based on *F*^2^ are statistically about twice as large as those based on *F*, and *R*- factors based on ALL data will be even larger.

### Fractional atomic coordinates and isotropic or equivalent isotropic displacement parameters (Å^2^)

**Table:**

	*x*	*y*	*z*	*U*_iso_*/*U*_eq_	
Ni1	1.0000	0.5000	0.5000	0.0250 (2)	
Tb1	0.68072 (2)	0.07753 (2)	0.702898 (17)	0.01601 (8)	
C1	0.7020 (4)	0.4733 (4)	0.7262 (3)	0.0183 (11)	
C2	0.6789 (4)	0.4175 (4)	0.8277 (3)	0.0183 (11)	
H2	0.6736	0.3308	0.8459	0.022*	
C3	0.6638 (4)	0.4905 (4)	0.9018 (3)	0.0175 (11)	
C4	0.6652 (4)	0.6204 (4)	0.8744 (3)	0.0178 (10)	
H4	0.6536	0.6703	0.9236	0.021*	
C5	0.6839 (4)	0.6748 (4)	0.7733 (3)	0.0158 (10)	
C6	0.7036 (4)	0.6024 (4)	0.6993 (3)	0.0193 (11)	
H6	0.7179	0.6402	0.6317	0.023*	
C7	0.6867 (4)	0.8146 (4)	0.7468 (3)	0.0175 (10)	
C8	0.7334 (5)	0.3941 (4)	0.6453 (4)	0.0204 (11)	
C9	0.6652 (5)	0.4829 (5)	1.0811 (4)	0.0273 (12)	
C10	0.6518 (5)	0.4003 (5)	1.1831 (4)	0.0236 (12)	
C11	0.6510 (5)	0.2706 (5)	1.2016 (4)	0.0289 (13)	
H11	0.6537	0.2274	1.1489	0.035*	
C12	0.6461 (5)	0.2065 (5)	1.2977 (4)	0.0335 (14)	
H12	0.6461	0.1194	1.3084	0.040*	
C13	0.6395 (6)	0.3855 (6)	1.3606 (4)	0.0377 (14)	
H13	0.6341	0.4257	1.4154	0.045*	
C14	0.6452 (5)	0.4584 (5)	1.2657 (4)	0.0338 (13)	
H14	0.6446	0.5454	1.2574	0.041*	
C15	1.0258 (4)	0.1208 (4)	0.8319 (3)	0.0182 (11)	
C16	1.1580 (5)	0.1051 (4)	0.7839 (3)	0.0202 (11)	
H16	1.1759	0.0885	0.7186	0.024*	
C17	1.2619 (4)	0.1142 (4)	0.8325 (3)	0.0188 (11)	
C18	1.2340 (5)	0.1428 (4)	0.9301 (3)	0.0199 (11)	
H18	1.3034	0.1484	0.9636	0.024*	
C19	1.1034 (5)	0.1627 (4)	0.9764 (3)	0.0202 (11)	
C20	0.9994 (5)	0.1486 (4)	0.9280 (3)	0.0202 (11)	
H20	0.9116	0.1578	0.9605	0.024*	
C21	0.9089 (5)	0.1091 (4)	0.7832 (4)	0.0190 (11)	
C22	1.4064 (5)	0.0981 (4)	0.7846 (4)	0.0209 (11)	
C23	0.9939 (5)	0.2964 (5)	1.0918 (4)	0.0243 (12)	
C24	0.9908 (5)	0.3390 (5)	1.1902 (4)	0.0239 (12)	
C25	0.9825 (5)	0.2574 (5)	1.2813 (4)	0.0249 (12)	
H25	0.9818	0.1711	1.2847	0.030*	
C26	0.9751 (5)	0.3078 (5)	1.3671 (4)	0.0259 (12)	
H26	0.9668	0.2531	1.4287	0.031*	
C27	0.9848 (5)	0.5064 (5)	1.2781 (4)	0.0260 (12)	
H27	0.9863	0.5924	1.2762	0.031*	
C28	0.9886 (5)	0.4653 (5)	1.1899 (4)	0.0254 (12)	
H28	0.9897	0.5224	1.1301	0.031*	
N1	0.6471 (4)	0.4307 (4)	1.0036 (3)	0.0198 (9)	
H1	0.6233	0.3539	1.0175	0.024*	
N2	0.6415 (4)	0.2613 (4)	1.3772 (3)	0.0351 (12)	
N3	1.0796 (4)	0.2002 (4)	1.0719 (3)	0.0215 (9)	
H3	1.1205	0.1609	1.1182	0.026*	
N4	0.9792 (4)	0.4297 (4)	1.3670 (3)	0.0230 (10)	
O1	0.8077 (3)	0.4367 (3)	0.5645 (2)	0.0279 (8)	
O2	0.6811 (3)	0.2878 (3)	0.6642 (2)	0.0299 (9)	
O3	0.6713 (4)	0.8705 (3)	0.6617 (2)	0.0331 (9)	
O4	0.7068 (4)	0.8772 (3)	0.8101 (2)	0.0339 (9)	
O5	0.6912 (5)	0.5911 (4)	1.0737 (3)	0.0543 (13)	
O6	0.9267 (3)	0.0947 (3)	0.6916 (2)	0.0292 (9)	
O7	0.7926 (3)	0.1150 (3)	0.8333 (2)	0.0223 (8)	
O8	1.4397 (3)	0.0991 (3)	0.6916 (2)	0.0279 (8)	
O9	1.4954 (3)	0.0850 (4)	0.8377 (2)	0.0389 (10)	
O10	0.9241 (4)	0.3510 (4)	1.0333 (3)	0.0367 (10)	
O1W	0.9258 (3)	0.6831 (3)	0.4332 (2)	0.0304 (9)	
H1WB	0.8653	0.7199	0.4703	0.036*	
H1WA	0.9887	0.7246	0.3992	0.036*	
O2W	0.7196 (4)	0.0949 (4)	0.5260 (3)	0.0384 (10)	
H2WA	0.762 (6)	0.038 (5)	0.497 (5)	0.08 (3)*	
H2WB	0.6846	0.1625	0.4985	0.046*	
O3W	0.2999 (4)	0.1656 (5)	0.5331 (3)	0.0711 (15)	
H3WC	0.3276	0.1462	0.4754	0.085*	
H3WD	0.3569	0.1383	0.5705	0.085*	
O4W	0.4141 (5)	0.8386 (4)	0.9854 (3)	0.0655 (15)	
H4WA	0.4330	0.9020	0.9390	0.079*	
H4WB	0.4387	0.8527	1.0378	0.079*	

### Atomic displacement parameters (Å^2^)

**Table:**

	*U*^11^	*U*^22^	*U*^33^	*U*^12^	*U*^13^	*U*^23^
Ni1	0.0299 (6)	0.0268 (5)	0.0192 (5)	−0.0041 (4)	−0.0022 (4)	−0.0074 (4)
Tb1	0.01649 (14)	0.01550 (13)	0.01686 (13)	−0.00072 (9)	−0.00436 (9)	−0.00362 (9)
C1	0.018 (3)	0.017 (3)	0.019 (3)	−0.001 (2)	−0.004 (2)	−0.002 (2)
C2	0.019 (3)	0.015 (3)	0.017 (3)	−0.002 (2)	0.001 (2)	0.001 (2)
C3	0.011 (2)	0.023 (3)	0.016 (3)	0.001 (2)	−0.001 (2)	0.002 (2)
C4	0.015 (3)	0.020 (3)	0.019 (3)	0.001 (2)	−0.006 (2)	−0.004 (2)
C5	0.014 (2)	0.014 (2)	0.019 (3)	−0.0025 (19)	−0.003 (2)	−0.001 (2)
C6	0.019 (3)	0.020 (3)	0.017 (3)	−0.002 (2)	−0.001 (2)	0.000 (2)
C7	0.016 (3)	0.017 (3)	0.021 (3)	0.000 (2)	−0.005 (2)	−0.005 (2)
C8	0.021 (3)	0.018 (3)	0.024 (3)	0.004 (2)	−0.010 (2)	−0.003 (2)
C9	0.026 (3)	0.031 (3)	0.023 (3)	−0.002 (2)	−0.002 (2)	−0.001 (2)
C10	0.016 (3)	0.030 (3)	0.023 (3)	−0.002 (2)	−0.004 (2)	−0.001 (2)
C11	0.034 (3)	0.028 (3)	0.025 (3)	−0.006 (2)	−0.009 (3)	−0.002 (2)
C12	0.038 (3)	0.030 (3)	0.032 (3)	−0.011 (3)	−0.011 (3)	0.005 (3)
C13	0.046 (4)	0.052 (4)	0.018 (3)	0.005 (3)	−0.008 (3)	−0.012 (3)
C14	0.040 (4)	0.034 (3)	0.027 (3)	−0.002 (3)	−0.007 (3)	−0.004 (3)
C15	0.017 (3)	0.019 (3)	0.020 (3)	0.001 (2)	−0.006 (2)	−0.004 (2)
C16	0.022 (3)	0.024 (3)	0.015 (2)	0.001 (2)	−0.003 (2)	−0.006 (2)
C17	0.013 (3)	0.024 (3)	0.020 (3)	−0.001 (2)	−0.005 (2)	−0.002 (2)
C18	0.017 (3)	0.022 (3)	0.024 (3)	−0.003 (2)	−0.011 (2)	−0.007 (2)
C19	0.020 (3)	0.024 (3)	0.020 (3)	−0.001 (2)	−0.003 (2)	−0.010 (2)
C20	0.016 (3)	0.024 (3)	0.022 (3)	0.001 (2)	−0.002 (2)	−0.010 (2)
C21	0.022 (3)	0.016 (3)	0.022 (3)	−0.001 (2)	−0.008 (2)	−0.006 (2)
C22	0.022 (3)	0.017 (3)	0.024 (3)	0.000 (2)	−0.004 (2)	−0.003 (2)
C23	0.020 (3)	0.032 (3)	0.023 (3)	−0.005 (2)	−0.002 (2)	−0.012 (2)
C24	0.014 (3)	0.039 (3)	0.020 (3)	0.000 (2)	−0.004 (2)	−0.010 (2)
C25	0.024 (3)	0.032 (3)	0.022 (3)	−0.002 (2)	−0.005 (2)	−0.011 (2)
C26	0.028 (3)	0.029 (3)	0.019 (3)	−0.009 (2)	−0.003 (2)	−0.001 (2)
C27	0.027 (3)	0.025 (3)	0.026 (3)	−0.002 (2)	−0.002 (2)	−0.006 (2)
C28	0.029 (3)	0.032 (3)	0.016 (3)	0.001 (2)	−0.005 (2)	−0.005 (2)
N1	0.024 (2)	0.016 (2)	0.017 (2)	−0.0011 (17)	−0.0026 (18)	0.0018 (17)
N2	0.041 (3)	0.040 (3)	0.023 (3)	−0.010 (2)	−0.009 (2)	0.004 (2)
N3	0.020 (2)	0.030 (2)	0.016 (2)	0.0027 (18)	−0.0079 (18)	−0.0040 (18)
N4	0.020 (2)	0.034 (3)	0.017 (2)	−0.0042 (19)	−0.0025 (18)	−0.0100 (19)
O1	0.028 (2)	0.037 (2)	0.0188 (19)	−0.0097 (17)	0.0015 (16)	−0.0089 (16)
O2	0.046 (2)	0.0134 (19)	0.030 (2)	−0.0048 (16)	−0.0039 (18)	−0.0058 (15)
O3	0.068 (3)	0.0138 (19)	0.0196 (19)	−0.0010 (17)	−0.0143 (19)	−0.0012 (15)
O4	0.067 (3)	0.0150 (19)	0.028 (2)	0.0044 (17)	−0.027 (2)	−0.0068 (15)
O5	0.111 (4)	0.026 (2)	0.031 (2)	−0.024 (2)	−0.023 (2)	−0.0010 (18)
O6	0.019 (2)	0.050 (2)	0.023 (2)	−0.0029 (17)	−0.0051 (16)	−0.0159 (17)
O7	0.0126 (18)	0.035 (2)	0.0232 (19)	−0.0012 (15)	−0.0038 (15)	−0.0134 (15)
O8	0.0151 (19)	0.050 (2)	0.022 (2)	0.0009 (16)	−0.0038 (15)	−0.0162 (17)
O9	0.0142 (19)	0.082 (3)	0.021 (2)	0.0050 (19)	−0.0062 (16)	−0.0094 (19)
O10	0.038 (2)	0.050 (3)	0.029 (2)	0.0195 (19)	−0.0152 (19)	−0.0220 (18)
O1W	0.035 (2)	0.031 (2)	0.0230 (19)	0.0021 (17)	0.0013 (17)	−0.0064 (16)
O2W	0.064 (3)	0.030 (2)	0.018 (2)	0.004 (2)	−0.009 (2)	0.0003 (17)
O3W	0.059 (3)	0.124 (5)	0.027 (2)	0.027 (3)	−0.013 (2)	−0.012 (3)
O4W	0.137 (5)	0.035 (3)	0.027 (2)	−0.031 (3)	−0.014 (3)	−0.0027 (19)

### Geometric parameters (Å, º)

**Table:**

Ni1—O1	2.096 (3)	C15—C20	1.383 (6)
Ni1—N4^i^	2.161 (4)	C15—C16	1.399 (6)
Ni1—O1W	2.181 (3)	C15—C21	1.504 (6)
Tb1—O2	2.245 (3)	C16—C17	1.379 (6)
Tb1—O2W	2.359 (4)	C16—H16	0.9300
Tb1—O9^ii^	2.388 (3)	C17—C18	1.405 (6)
Tb1—O7	2.413 (3)	C17—C22	1.511 (6)
Tb1—O4^iii^	2.416 (3)	C18—C19	1.386 (6)
Tb1—O3^iii^	2.433 (3)	C18—H18	0.9300
Tb1—O8^ii^	2.495 (3)	C19—C20	1.392 (6)
Tb1—O6	2.509 (3)	C19—N3	1.418 (6)
C1—C6	1.381 (6)	C20—H20	0.9300
C1—C2	1.392 (6)	C21—O7	1.257 (5)
C1—C8	1.506 (6)	C21—O6	1.275 (5)
C2—C3	1.385 (6)	C22—O8	1.255 (5)
C2—H2	0.9300	C22—O9	1.259 (6)
C3—C4	1.389 (6)	C23—O10	1.225 (6)
C3—N1	1.409 (6)	C23—N3	1.354 (6)
C4—C5	1.384 (6)	C23—C24	1.505 (7)
C4—H4	0.9300	C24—C28	1.370 (7)
C5—C6	1.377 (6)	C24—C25	1.381 (7)
C5—C7	1.493 (6)	C25—C26	1.379 (6)
C6—H6	0.9300	C25—H25	0.9300
C7—O3	1.246 (5)	C26—N4	1.327 (6)
C7—O4	1.255 (5)	C26—H26	0.9300
C8—O1	1.243 (5)	C27—N4	1.336 (6)
C8—O2	1.264 (5)	C27—C28	1.361 (7)
C9—O5	1.203 (6)	C27—H27	0.9300
C9—N1	1.345 (6)	C28—H28	0.9300
C9—C10	1.500 (7)	N1—H1	0.8600
C10—C11	1.384 (7)	N3—H3	0.8600
C10—C14	1.389 (7)	O1W—H1WB	0.8524
C11—C12	1.365 (7)	O1W—H1WA	0.8227
C11—H11	0.9300	O2W—H2WA	0.849 (5)
C12—N2	1.331 (7)	O2W—H2WB	0.8506
C12—H12	0.9300	O3W—H3WC	0.8494
C13—N2	1.324 (7)	O3W—H3WD	0.8540
C13—C14	1.384 (7)	O4W—H4WA	0.8486
C13—H13	0.9300	O4W—H4WB	0.8510
C14—H14	0.9300		
			
O1—Ni1—O1^iv^	180.000 (1)	O5—C9—C10	118.2 (5)
O1—Ni1—N4^i^	87.40 (13)	N1—C9—C10	117.6 (5)
O1^iv^—Ni1—N4^i^	92.60 (13)	C11—C10—C14	117.0 (5)
O1—Ni1—N4^v^	92.60 (13)	C11—C10—C9	125.5 (5)
O1^iv^—Ni1—N4^v^	87.40 (13)	C14—C10—C9	117.4 (5)
N4^i^—Ni1—N4^v^	180.000 (1)	C12—C11—C10	119.5 (5)
O1—Ni1—O1W	92.84 (13)	C12—C11—H11	120.3
O1^iv^—Ni1—O1W	87.16 (13)	C10—C11—H11	120.3
N4^i^—Ni1—O1W	88.99 (14)	N2—C12—C11	123.9 (5)
N4^v^—Ni1—O1W	91.01 (14)	N2—C12—H12	118.0
O1—Ni1—O1W^iv^	87.16 (13)	C11—C12—H12	118.0
O1^iv^—Ni1—O1W^iv^	92.84 (13)	N2—C13—C14	123.1 (5)
N4^i^—Ni1—O1W^iv^	91.01 (14)	N2—C13—H13	118.4
N4^v^—Ni1—O1W^iv^	88.99 (14)	C14—C13—H13	118.4
O1W—Ni1—O1W^iv^	180.00 (17)	C13—C14—C10	119.4 (5)
O2—Tb1—O2W	82.73 (13)	C13—C14—H14	120.3
O2—Tb1—O9^ii^	90.98 (13)	C10—C14—H14	120.3
O2W—Tb1—O9^ii^	138.08 (13)	C20—C15—C16	119.6 (4)
O2—Tb1—O7	81.89 (12)	C20—C15—C21	117.5 (4)
O2W—Tb1—O7	139.50 (13)	C16—C15—C21	122.8 (4)
O9^ii^—Tb1—O7	79.42 (11)	C17—C16—C15	120.7 (4)
O2—Tb1—O4^iii^	154.16 (12)	C17—C16—H16	119.7
O2W—Tb1—O4^iii^	120.54 (13)	C15—C16—H16	119.7
O9^ii^—Tb1—O4^iii^	78.80 (13)	C16—C17—C18	119.3 (4)
O7—Tb1—O4^iii^	72.98 (11)	C16—C17—C22	123.0 (4)
O2—Tb1—O3^iii^	152.82 (12)	C18—C17—C22	117.7 (4)
O2W—Tb1—O3^iii^	70.93 (12)	C19—C18—C17	120.2 (4)
O9^ii^—Tb1—O3^iii^	103.96 (13)	C19—C18—H18	119.9
O7—Tb1—O3^iii^	122.74 (11)	C17—C18—H18	119.9
O4^iii^—Tb1—O3^iii^	52.81 (11)	C18—C19—C20	119.8 (4)
O2—Tb1—O8^ii^	85.77 (12)	C18—C19—N3	118.6 (4)
O2W—Tb1—O8^ii^	85.49 (13)	C20—C19—N3	121.5 (4)
O9^ii^—Tb1—O8^ii^	52.66 (11)	C15—C20—C19	120.3 (4)
O7—Tb1—O8^ii^	130.18 (10)	C15—C20—H20	119.8
O4^iii^—Tb1—O8^ii^	105.78 (12)	C19—C20—H20	119.8
O3^iii^—Tb1—O8^ii^	85.57 (12)	O7—C21—O6	119.9 (4)
O2—Tb1—O6	84.52 (12)	O7—C21—C15	119.5 (4)
O2W—Tb1—O6	88.54 (13)	O6—C21—C15	120.6 (4)
O9^ii^—Tb1—O6	132.24 (11)	O8—C22—O9	119.1 (4)
O7—Tb1—O6	52.86 (10)	O8—C22—C17	120.7 (4)
O4^iii^—Tb1—O6	85.04 (12)	O9—C22—C17	120.1 (4)
O3^iii^—Tb1—O6	101.01 (12)	O8—C22—Tb1^vii^	62.0 (2)
O8^ii^—Tb1—O6	169.17 (11)	O9—C22—Tb1^vii^	57.1 (2)
O2—Tb1—C7^iii^	178.33 (13)	C17—C22—Tb1^vii^	176.4 (3)
O2W—Tb1—C7^iii^	96.05 (14)	O10—C23—N3	123.5 (5)
O9^ii^—Tb1—C7^iii^	90.69 (14)	O10—C23—C24	119.8 (5)
O7—Tb1—C7^iii^	98.38 (12)	N3—C23—C24	116.6 (5)
O4^iii^—Tb1—C7^iii^	26.52 (12)	C28—C24—C25	118.6 (5)
O3^iii^—Tb1—C7^iii^	26.32 (12)	C28—C24—C23	118.2 (4)
O8^ii^—Tb1—C7^iii^	95.29 (12)	C25—C24—C23	123.1 (5)
O6—Tb1—C7^iii^	94.32 (12)	C26—C25—C24	117.9 (5)
O2—Tb1—C22^ii^	87.76 (13)	C26—C25—H25	121.0
O2W—Tb1—C22^ii^	111.81 (15)	C24—C25—H25	121.0
O9^ii^—Tb1—C22^ii^	26.29 (12)	N4—C26—C25	124.0 (5)
O7—Tb1—C22^ii^	104.77 (12)	N4—C26—H26	118.0
O4^iii^—Tb1—C22^ii^	92.83 (13)	C25—C26—H26	118.0
O3^iii^—Tb1—C22^ii^	95.67 (13)	N4—C27—C28	123.4 (5)
O8^ii^—Tb1—C22^ii^	26.37 (12)	N4—C27—H27	118.3
O6—Tb1—C22^ii^	157.14 (13)	C28—C27—H27	118.3
C7^iii^—Tb1—C22^ii^	93.76 (13)	C27—C28—C24	119.4 (5)
C6—C1—C2	119.8 (4)	C27—C28—H28	120.3
C6—C1—C8	119.5 (4)	C24—C28—H28	120.3
C2—C1—C8	120.6 (4)	C9—N1—C3	125.9 (4)
C3—C2—C1	120.4 (4)	C9—N1—H1	117.1
C3—C2—H2	119.8	C3—N1—H1	117.1
C1—C2—H2	119.8	C13—N2—C12	117.1 (5)
C2—C3—C4	119.5 (4)	C23—N3—C19	121.5 (4)
C2—C3—N1	118.9 (4)	C23—N3—H3	119.3
C4—C3—N1	121.6 (4)	C19—N3—H3	119.3
C5—C4—C3	119.5 (4)	C26—N4—C27	116.7 (4)
C5—C4—H4	120.3	C26—N4—Ni1^viii^	121.8 (3)
C3—C4—H4	120.3	C27—N4—Ni1^viii^	121.1 (3)
C6—C5—C4	121.1 (4)	C8—O1—Ni1	144.0 (3)
C6—C5—C7	121.0 (4)	C8—O2—Tb1	154.9 (3)
C4—C5—C7	117.9 (4)	C7—O3—Tb1^vi^	93.7 (3)
C5—C6—C1	119.6 (4)	C7—O4—Tb1^vi^	94.2 (3)
C5—C6—H6	120.2	C21—O6—Tb1	91.1 (3)
C1—C6—H6	120.2	C21—O7—Tb1	96.0 (3)
O3—C7—O4	119.1 (4)	C22—O8—Tb1^vii^	91.6 (3)
O3—C7—C5	120.9 (4)	C22—O9—Tb1^vii^	96.6 (3)
O4—C7—C5	120.0 (4)	Ni1—O1W—H1WB	117.5
O3—C7—Tb1^vi^	60.0 (2)	Ni1—O1W—H1WA	109.4
O4—C7—Tb1^vi^	59.2 (2)	H1WB—O1W—H1WA	118.0
C5—C7—Tb1^vi^	177.4 (3)	Tb1—O2W—H2WA	122 (5)
O1—C8—O2	124.1 (4)	Tb1—O2W—H2WB	111.0
O1—C8—C1	118.7 (4)	H2WA—O2W—H2WB	127.1
O2—C8—C1	117.2 (4)	H3WC—O3W—H3WD	108.6
O5—C9—N1	124.2 (5)	H4WA—O4W—H4WB	107.8
			
C6—C1—C2—C3	−3.1 (7)	O5—C9—N1—C3	4.3 (8)
C8—C1—C2—C3	173.3 (4)	C10—C9—N1—C3	−175.7 (4)
C1—C2—C3—C4	3.2 (7)	C2—C3—N1—C9	161.9 (5)
C1—C2—C3—N1	−176.9 (4)	C4—C3—N1—C9	−18.2 (7)
C2—C3—C4—C5	−1.1 (7)	C14—C13—N2—C12	−1.5 (8)
N1—C3—C4—C5	179.0 (4)	C11—C12—N2—C13	1.0 (8)
C3—C4—C5—C6	−1.2 (7)	O10—C23—N3—C19	5.8 (7)
C3—C4—C5—C7	−179.1 (4)	C24—C23—N3—C19	−171.8 (4)
C4—C5—C6—C1	1.3 (7)	C18—C19—N3—C23	133.0 (5)
C7—C5—C6—C1	179.2 (4)	C20—C19—N3—C23	−45.5 (7)
C2—C1—C6—C5	0.8 (7)	C25—C26—N4—C27	3.1 (7)
C8—C1—C6—C5	−175.6 (4)	C25—C26—N4—Ni1^viii^	−169.7 (4)
C6—C5—C7—O3	19.3 (7)	C28—C27—N4—C26	−1.1 (7)
C4—C5—C7—O3	−162.8 (4)	C28—C27—N4—Ni1^viii^	171.7 (4)
C6—C5—C7—O4	−159.6 (4)	O2—C8—O1—Ni1	−119.0 (5)
C4—C5—C7—O4	18.3 (7)	C1—C8—O1—Ni1	61.6 (7)
C6—C1—C8—O1	27.8 (7)	N4^i^—Ni1—O1—C8	148.5 (6)
C2—C1—C8—O1	−148.5 (5)	N4^v^—Ni1—O1—C8	−31.5 (6)
C6—C1—C8—O2	−151.7 (4)	O1W—Ni1—O1—C8	−122.6 (6)
C2—C1—C8—O2	32.0 (7)	O1W^iv^—Ni1—O1—C8	57.4 (6)
O5—C9—C10—C11	−165.2 (5)	O1—C8—O2—Tb1	74.5 (9)
N1—C9—C10—C11	14.7 (8)	C1—C8—O2—Tb1	−106.0 (7)
O5—C9—C10—C14	12.0 (8)	O2W—Tb1—O2—C8	−92.7 (8)
N1—C9—C10—C14	−168.1 (5)	O9^ii^—Tb1—O2—C8	128.9 (8)
C14—C10—C11—C12	−1.1 (8)	O7—Tb1—O2—C8	49.7 (7)
C9—C10—C11—C12	176.1 (5)	O4^iii^—Tb1—O2—C8	63.1 (8)
C10—C11—C12—N2	0.3 (8)	O3^iii^—Tb1—O2—C8	−107.0 (8)
N2—C13—C14—C10	0.7 (9)	O8^ii^—Tb1—O2—C8	−178.7 (8)
C11—C10—C14—C13	0.6 (8)	O6—Tb1—O2—C8	−3.5 (7)
C9—C10—C14—C13	−176.8 (5)	C22^ii^—Tb1—O2—C8	154.9 (8)
C20—C15—C16—C17	1.6 (7)	O4—C7—O3—Tb1^vi^	−3.9 (5)
C21—C15—C16—C17	−178.7 (4)	C5—C7—O3—Tb1^vi^	177.1 (4)
C15—C16—C17—C18	−1.7 (7)	O3—C7—O4—Tb1^vi^	4.0 (5)
C15—C16—C17—C22	179.9 (4)	C5—C7—O4—Tb1^vi^	−177.1 (4)
C16—C17—C18—C19	−0.6 (7)	O7—C21—O6—Tb1	−3.3 (4)
C22—C17—C18—C19	177.9 (4)	C15—C21—O6—Tb1	177.3 (4)
C17—C18—C19—C20	3.0 (7)	O2—Tb1—O6—C21	85.9 (3)
C17—C18—C19—N3	−175.6 (4)	O2W—Tb1—O6—C21	168.7 (3)
C16—C15—C20—C19	0.9 (7)	O9^ii^—Tb1—O6—C21	−0.5 (3)
C21—C15—C20—C19	−178.9 (4)	O7—Tb1—O6—C21	1.9 (2)
C18—C19—C20—C15	−3.2 (7)	O4^iii^—Tb1—O6—C21	−70.5 (3)
N3—C19—C20—C15	175.3 (4)	O3^iii^—Tb1—O6—C21	−121.1 (3)
C20—C15—C21—O7	−5.5 (7)	O8^ii^—Tb1—O6—C21	112.2 (6)
C16—C15—C21—O7	174.7 (4)	C7^iii^—Tb1—O6—C21	−95.3 (3)
C20—C15—C21—O6	173.8 (4)	C22^ii^—Tb1—O6—C21	15.1 (5)
C16—C15—C21—O6	−6.0 (7)	O6—C21—O7—Tb1	3.5 (5)
C16—C17—C22—O8	13.6 (7)	C15—C21—O7—Tb1	−177.2 (4)
C18—C17—C22—O8	−164.8 (4)	O2—Tb1—O7—C21	−91.2 (3)
C16—C17—C22—O9	−167.3 (5)	O2W—Tb1—O7—C21	−22.5 (4)
C18—C17—C22—O9	14.3 (7)	O9^ii^—Tb1—O7—C21	176.3 (3)
O10—C23—C24—C28	−41.8 (7)	O4^iii^—Tb1—O7—C21	94.9 (3)
N3—C23—C24—C28	136.0 (5)	O3^iii^—Tb1—O7—C21	76.4 (3)
O10—C23—C24—C25	134.5 (5)	O8^ii^—Tb1—O7—C21	−168.6 (3)
N3—C23—C24—C25	−47.8 (7)	O6—Tb1—O7—C21	−1.9 (3)
C28—C24—C25—C26	−1.3 (7)	C7^iii^—Tb1—O7—C21	87.1 (3)
C23—C24—C25—C26	−177.5 (4)	C22^ii^—Tb1—O7—C21	−176.7 (3)
C24—C25—C26—N4	−1.9 (8)	O9—C22—O8—Tb1^vii^	−1.7 (5)
N4—C27—C28—C24	−2.0 (8)	C17—C22—O8—Tb1^vii^	177.4 (4)
C25—C24—C28—C27	3.2 (7)	O8—C22—O9—Tb1^vii^	1.8 (5)
C23—C24—C28—C27	179.5 (4)	C17—C22—O9—Tb1^vii^	−177.3 (4)

Symmetry codes: (i) *x*, *y*, *z*−1; (ii) *x*−1, *y*, *z*; (iii) *x*, *y*−1, *z*; (iv) −*x*+2, −*y*+1, −*z*+1; (v) −*x*+2, −*y*+1, −*z*+2; (vi) *x*, *y*+1, *z*; (vii) *x*+1, *y*, *z*; (viii) *x*, *y*, *z*+1.

### Hydrogen-bond geometry (Å, º)

**Table:**

*D*—H···*A*	*D*—H	H···*A*	*D*···*A*	*D*—H···*A*
N1—H1···O4*W*^ix^	0.86	2.16	3.000 (6)	166
N3—H3···O4^v^	0.86	2.17	2.958 (6)	153
O1*W*—H1*WA*···O6^iv^	0.82	2.24	2.988 (4)	151
O1*W*—H1*WB*···O3*W*^x^	0.85	2.04	2.763 (6)	143
O2*W*—H2*WA*···O3*W*^xi^	0.85 (6)	2.47 (6)	3.117 (7)	134 (5)
O2*W*—H2*WB*···N2^i^	0.85	1.92	2.672 (6)	147
O3*W*—H3*WC*···O3^x^	0.85	1.92	2.736 (5)	159
O3*W*—H3*WD*···O8^ii^	0.85	1.98	2.793 (5)	160
O4*W*—H4*WA*···O9^xii^	0.85	2.25	3.088 (6)	170
O4*W*—H4*WB*···O9^v^	0.85	2.19	3.034 (5)	172

Symmetry codes: (i) *x*, *y*, *z*−1; (ii) *x*−1, *y*, *z*; (iv) −*x*+2, −*y*+1, −*z*+1; (v) −*x*+2, −*y*+1, −*z*+2; (ix) −*x*+1, −*y*+1, −*z*+2; (x) −*x*+1, −*y*+1, −*z*+1; (xi) −*x*+1, −*y*, −*z*+1; (xii) *x*−1, *y*+1, *z*.

## References

[bb1] Brandenburg, K. (1999). *DIAMOND* Crystal Impact GbR, Bonn, Germany.

[bb2] Bruker (2001). *SADABS* Bruker AXS Inc., Madison, Wisconsin, USA.

[bb3] Bruker (2007). *SMART* and *SAINT* Bruker AXS Inc., Madison, Wisconsin, USA.

[bb4] Chen, M.-S., Zhao, Y., Okamura, T.-A., Su, Z., Sun, W.-Y. & Ueyama, N. (2011). *Supramol. Chem.* **23**, 117–124.

[bb5] Deng, Y.-F., Chen, M.-S., Zhang, C.-H. & Kuang, D.-Z. (2011). *Acta Cryst.* E**67**, m1431–m1432. 10.1107/S1600536811038074 PMC3201412 22064912

[bb6] Gu, X.-J. & Xue, D.-F. (2006). *Inorg. Chem.* **45**, 9257–9261. 10.1021/ic060806l 17083224

[bb7] Liang, Y.-C., Cao, R., Su, W.-P., Hong, M.-C. & Zhang, W.-J. (2000). *Angew. Chem. Int. Ed.* **39**, 3304–3307. 10.1002/1521-3773(20000915)39:18<3304::aid-anie3304>3.0.co;2-h 11028085

[bb8] Prasad, T. K., Rajasekharan, M. V. & Costes, J. P. (2007). *Angew. Chem. Int. Ed.* **46**, 2851–2854. 10.1002/anie.200605062 17351996

[bb9] Sheldrick, G. M. (2008). *Acta Cryst.* A**64**, 112–122. 10.1107/S0108767307043930 18156677

[bb10] Zhao, B., Cheng, P., Chen, X.-Y., Cheng, C., Shi, W., Liao, D.-Z., Yan, S.-P. & Jiang, Z.-H. (2004). *J. Am. Chem. Soc.* **126**, 3012–3013. 10.1021/ja038784e 15012106

[bb11] Zhao, B., Cheng, P., Dai, Y., Cheng, C., Liao, D.-Z., Yan, S.-P., Jiang, Z.-H. & Wang, G.-L. (2003). *Angew. Chem. Int. Ed.* **42**, 934–936. 10.1002/anie.200390248 12596182

